# Association of *CYP19A1* and *CYP1A2* genetic polymorphisms with type 2 diabetes mellitus risk in the Chinese Han population

**DOI:** 10.1186/s12944-020-01366-9

**Published:** 2020-08-19

**Authors:** Yafeng Yang, Ping Wang

**Affiliations:** 1 Department of clinical nutrition, Xianyang Central Hospital, Xianyang, 712000 Shaanxi Province China; 2 Department of hemodialysis, Xianyang Central Hospital, Xianyang, 712000 Shaanxi Province China

**Keywords:** *CYP19A1*, *CYP1A2*, Type 2 diabetes mellitus, Single nucleotide polymorphism

## Abstract

**Background:**

Type 2 diabetes mellitus (T2DM), one of the global health issues, is a group of metabolic diseases and is affected by several genetic loci in the clinical phenotype. This study intended to ascertain associations between *CYP19A1* and *CYP1A2* gene polymorphisms with the T2DM risk in Chinese Han.

**Methods:**

Seven single nucleotide polymorphisms (SNPs) in total including five of *CYP19A1* (rs4646, rs6493487, rs1062033, rs17601876 and rs3751599) and two of *CYP1A2* (rs762551 and rs2470890) from 512 T2DM patients and 515 non-diabetic controls were genotyped in the platform of Agena MassARRAY. SPSS 18.0 was utilized for analyzing genotyping results. Logistic regression models were conducted for the risk assessment by the odds ratios (ORs) and 95% confidence intervals (CIs).

**Results:**

The results suggested a significant association between genotype GC of rs1062033 with a decreased T2DM risk (OR = 0.73, 95% CI = 0.55–0.96, *P* = 0.025) under the co-dominant (heterozygous) model. The results of stratification analysis with age and gender adjustment revealed that the effects of all selected SNPs in *CYP19A1* and *CYP1A2* on the T2DM susceptibility were dependent on age, body mass index (BMI) and disease progression (*P* <  0.05). The haplotype analysis was further conducted and the results indicated that C_rs1062033_G_rs17601876_A_rs3751599_ in *CYP19A1* played a protective role (OR = 0.48, 95% CI = 0.25–0.91, *P* = 0.026) in T2DM patients with diabetic retinopathy.

**Conclusion:**

This population-based case-control study suggested that *CYP19A1* and *CYP1A2* variations might affect the susceptibility of T2DM. The findings provide a theoretical basis for searching the clinical therapeutic markers and attractive drug targets of T2DM.

## Introduction

Type 2 diabetes mellitus (T2DM) is a kind of chronic metabolic diseases characterized by chronic hyperglycemia arising from insulin secretion disorders, and/or the insulin resistance [1–3]. Besides, genetic predisposition, sedentary lifestyle, and excessive calorie intake may also contribute to T2DM [4]. Studies have shown that the incidence of diabetes is related to genetic factors and is the result of the joint action of multiple gene loci [4, 5]. Genome-wide association studies (GWAS), genome-wide linkage analysis and candidate-gene approaches are wildly applied in studying the genetic basis of T2DM [1, 3]. To date, multiple genes have been functionally implicated in the pathogenesis of T2DM [1, 4]. However, the susceptibility of T2DM varies across populations due to differences in interracial gene polymorphisms and haplotypes. Therefore, it is of great significance to expand the studies of T2DM susceptibility genes in different populations.

*CYP19A1* locates at 15q21.2 containing 10 exons and spanning a 130-kb region, and encodes the aromatase enzyme which is associated with changes in aromatase levels [6]. Aromatase plays a crucial role in the final stage of estrogen biosynthesis, and can be affected by genetic factors resulting in the changes in serum sex hormone levels [7, 8], and recent evidences indicated that the rs4646 variant of *CYP19A1* might be a predictive factor of the benefit of aromatase inhibitor treatment for breast cancer [9]. Meanwhile, *CYP19A1* gene polymorphisms were considered to be related with coronary artery disease and circulating sex hormone levels in Chinese Uyghurs [10]. *CYP19A1* was also found to be associated with cardiovascular risk factors such as insulin resistance and hypertension in a sex- and obesity- specific manner [11]. Recently, a case-control study on associations between *CYP19A1* polymorphisms and obesity in Turkish population illustrated that the reduced aromatase activity is a risk factor for obesity, and *CYP19A1* is associated with hyperandrogenism which may play a role in abdominal obesity pathogenesis [12]. The above studies confirmed that *CYP19A1* may be associated with diabetic-related diseases. However, the relationship between *CYP19A1* polymorphisms and the T2DM susceptibility has not been reported, especially in the population of Chinese Han.

*CYP1A2* locates at 15q24.1 containing seven exons and six introns and spanning a 78-kb region, and encodes monooxygenase that can catalyze many reactions involved in drug metabolisms and synthesis of cholesterol, steroids and other lipids [13–15]. *CYP1A2* is mainly expressed in mammalian liver, and participates in the metabolisms of over 100 substrates [13, 16]. Genetic polymorphisms of *CYP1A2* have been extensively studied in a variety of populations, and were found to be involved in the etiology of developing cancers and other diseases [13, 16–19]. Moreover, a related study on CYP450 activities proved that the activity of *CYP1A2* is slightly increased in the subjects with diabetes [20]. Previous studies also demonstrated that the enzyme activity of *CYP1A2* and the speed of caffeine metabolism are increased in the T2DM group because of a higher caffeine intake [21]. The gene expression of *CYP1A2* was also found to decrease in mice fed a high-fat diet [22]. In all above mentioned studies, researches have examined the functional activity of *CYP1A2* in T2DM, but none of them focused on the genetic association.

To further investigate the role of *CYP19A1* and *CYP1A2* variations in the T2DM risk, this case-control study was set up to genotype 7 single nucleotide polymorphisms (SNPs) of T2DM patients and non-diabetic controls from the population of Chinese Han. The purpose of this study is to better understand the relationship between the population characteristics and the susceptibility to T2DM at the genetic level, and provide valuable diagnostic markers or targeted drug therapy strategies for T2DM by studying gene polymorphisms in the population of Chinese.

## Methods

### Study population

This case-control study involved 1027 participants comprising 512 patients with T2DM (54.9% males, mean age 59.2 ± 9.6 years) and 515 non-diabetic controls (55.0% males, mean age 59.3 ± 11.0 years) from the population of Chinese Han. Patients with T2DM were diagnosed according to the World Health Organization criteria (fast plasma glucose (FPG) ≥ 7.0 mmol/L and/or 2-h plasma glucose ≥11.1 mmol/L) and were recruited from September 2017 to June 2019. Cases with other diabetic types or treated by drugs (except anti-diabetic drugs) were excluded. The non-diabetic control subjects had normal glucose tolerance confirmed by FPG ≤ 6.0 mmol/L, or HbA1c levels < 6.5%, and had no history of diabetes in first or second degree relatives. All participants signed the informed consents and authorization for blood sampling and banking. This study fully complied with the standard of Helsinki declaration and was permitted by the ethics committee of the First Affiliated Hospital of Xi’an Jiaotong University.

### SNP selection and genotyping

Genomic DNA extraction was performed on the basis of the manufacturer’s procedures of GoldMag Beads DNA Extraction Kit (GoldMag, Xi’an, Shaanxi, China). DNA concentration was determined by Spectrometry (Beckman Instruments, Fullerton, CA, USA). Agena MassARRAY platform (Agena Bioscience, San Diego, CA, USA) was utilized for SNP genotyping. The design of extended primers was conducted by the Agena online design software (https://agenacx.com/online-tools/), and the sequences were listed in Supplementary Table 1. The process of genotyping was double-blinded by two laboratory personnel. For quality control, 10% of the samples were randomly chosen for repeated genotyping, and the reproducibility was 100%.

All seven candidate SNPs in *CYP19A1* (rs4646, rs6493487, rs1062033, rs17601876 and rs3751599) and *CYP1A2* (rs762551 and rs2470890) were screened using the database of dbSNP in NCBI and the 1000 Genomes Project data, and the selection criteria were as follows: i) the minor allele frequency (MAF) of all SNPs was greater than 5%; ii) call rate was over 95% during genotyping; iii) r^2^, a pairwise linkage disequilibrium (LD), was over 0.8. Besides, we applied RegulomeDB annotations to predict the effect of these SNPs according to the rank score evaluated by a model integrating functional genomic features.

### Statistical analysis

The acquired data was statistically analyzed by SPSS 18.0 (SPSS, Chicago, IL, USA) and PLINK 1.07 packages. Differences in clinical characteristics between cases and controls were analyzed by Welch’s t-test and Pearson’s chi-squared test where appropriate. The allele and genotype frequencies in cases and controls were calculated by χ^2^ test. Hardy-Weinberg equilibrium (HWE) for each SNP in the control group was determined by fisher’s exact test. Logistic analysis was performed to assess the correlation between the genetic variants and T2DM risk under allele, co-dominant, dominant, recessive and additive genetic models, respectively. Odds ratios (ORs) and 95% confidence intervals (CIs) from a logistic regression model were calculated after adjusting for age and gender. The *P* value (two-tailed) less than 0.05 indicated a statistical significance.

## Results

### Demographics of study subjects and genotypic characteristics of SNPs

In this study, seven SNPs of *CYP19A1* and *CYP1A2* from 512 T2DM patients and 515 non-diabetic controls were genotyped. The demographic details of the study subjects and the characteristic features of the studied SNPs were depicted in Tables 1 and 2, respectively. The frequency distributions of age (*P* = 0.990) and gender (*P* = 0.962) were matched between T2DM patients and non-diabetic controls.

**Table 1:** Demographics and clinical characteristics of cases and controls

Variables	Case (***n*** = 512)	Control (***n*** = 515)	***P***
Age	59.2 ± 9.6	59.3 ± 11.0	0.990^a^
≤ 59	248	243	
> 59	264	272	
Gender			0.962^b^
Male	281	283	
Female	231	232	
BMI			
≤ 24	130	126	
> 24	190	123	
Tobacco smoking status			
Yes	135	132	
No	231	137	
Alcohol consumption status			
Yes	69	98	
No	278	138	
Disease course			
≤ 9	151		
> 9	186		
Complication			
one	108		
multiple	141		
Antidiabetes drug			
Yes	128		
No	204		
Insulin			
Yes	175		
No	157		
Diabetic retinopathy			
Yes	213		
No	149		
FPG (mmol/L)	9.95 ± 4.69	5.67 ± 0.78	< 0.001^a*^
HbA1c (%)	9.30 ± 2.47	5.88 ± 0.79	0.004^a*^

The clinical information of participants was partially missing except age, gender and FPG/ HbA1c indexes

*BMI* body mass index, *FPG* fasting plasma glucose, *HbA1c* Hemoglobin A1c

^a^*P* was calculated by t test

^b^*P* was calculated by Pearson’s chi-squared test

*P < 0.05 indicates a significant difference

**Table 2:** Information and function annotation of SNPs in *CYP19A1* and *CYP1A2*

Gene	SNP	Chromosome	Position	Allele A/B	Role	MAF		***p***-HWE	ORs (95% CI)	***P***	RegulomeDB Rank
						Case	Control				
*CYP19A1*	rs4646	15	51,210,647	A/C	Intron	0.314	0.291	0.669	1.12(0.92–1.35)	0.257	5
*CYP19A1*	rs6493487	15	51,221,532	G/A	Intron	0.288	0.265	0.734	1.12(0.93–1.37)	0.236	6
*CYP19A1*	rs1062033	15	51,255,741	G/C	5′ UTR	0.432	0.448	0.110	0.94(0.79–1.12)	0.464	5
*CYP19A1*	rs17601876	15	51,261,712	A/G	Intron	0.341	0.334	0.921	1.03(0.86–1.24)	0.743	7
*CYP19A1*	rs3751599	15	51,281,336	A/G	Intron	0.058	0.074	1	1.07(0.54–1.09)	0.139	2b
*CYP1A2*	rs762551	15	74,749,576	C/A	Intron	0.402	0.414	0.928	0.95(0.80–1.14)	0.595	5
*CYP1A2*	rs2470890	15	74,755,085	T/C	Coding sequence	0.121	0.115	0.187	1.07(0.81–1.39)	0.646	1f

*SNP* single nucleotide polymorphism, *MAF* minor allele frequency, *HWE* Hardy-Weinberg Equilibrium, *OR* odds ratio, *95% CI* 95% confidence interval

Rank 1f, eQTL + TF binding / DNase peak; Rank 2b, TF binding + any motif + DNase Footprint + DNase peak; Rank 5, TF binding or DNase peak; Rank 6–7, other

All selected SNPs were consistent with HWE (*P* > 0.05), and the MAFs of those were larger than 0.05. The functional effects of SNPs were annotated by RegulomeDB, and were quantified by assigned rank scores accordingly. The results showed that rs2470890 of *CYP1A2* was likely to affect expression quantitative trait loci (eQTL) + transcription factor (TF) binding/DNase peak, and was assigned a rank score of 1f. *CYP19A1* rs3751599 was assigned a score of 2b and might affect TF binding + any motif + DNase Footprint + DNase peak. The other SNPs were assigned 5–7, and were likely to influence TF binding or DNase peak or others. The lower the grade score, the greater impacts on gene features and regulatory elements. The frequency distributions of the allele and genotype were shown in Supplementary Table 2, and allele “A” was defined as the minor allele. These data were used to compare the distribution differences of genotypes between cases and controls in the following analysis.

### Association of CYP19A1 and CYP1A2 polymorphisms with the T2DM susceptibility

To evaluate the allelic and genotypic distributions of all seven SNPs in *CYP19A1* and *CYP1A2*, inheritance models were established and the relevant results were organized in Table 3. Only GC genotype of rs1062033 in *CYP19A1* was detected to be significantly associated with a decreased risk of T2DM under the co-dominant (heterozygous) model (OR = 0.73, 95% CI = 0.55–0.96, *P* = 0.025). The association between the clinical indexes of T2DM and the *CYP19A1* rs1062033 polymorphism was further analyzed, but no significant correlation was observed (Supplementary Table 3).

**Table 3:** Association of *CYP19A1* and *CYP1A2* polymorphisms with T2DM risk

Gene	SNP	Model	Allele/Genotype	OR(95% CI)	***P***
*CYP19A1*	rs4646	Allele	A	1.12(0.92–1.35)	0.257
		Co-dominant (HOM)	AA vs CC	1.42(0.91–2.20)	0.121
		Co-dominant (HET)	AC vs CC	1.00(0.77–1.30)	0.982
		Dominant	AA-AC vs CC	1.07(0.84–1.37)	0.596
		Recessive	AA vs AC-CC	1.41(0.93–2.16)	0.109
		Additive		1.11(0.92–1.34)	0.260
*CYP19A1*	rs6493487	Allele	G	1.12(0.93–1.37)	0.236
		Co-dominant (HOM)	GG vs AA	1.52(0.95–2.42)	0.633
		Co-dominant (HET)	GA vs AA	0.99(0.76–1.28)	0.936
		Dominant	GG-GA vs AA	1.07(0.83–1.36)	0.616
		Recessive	GG vs GA-AA	1.52(0.97–2.40)	0.070
		Additive		1.12(0.93–1.36)	0.242
*CYP19A1*	rs1062033	Allele	G	0.94(0.79–1.12)	0.464
		Co-dominant (HOM)	GG vs CC	0.94(0.66–1.34)	0.736
		Co-dominant (HET)	GC vs CC	0.73(0.55–0.96)	0.025*
		Dominant	GG-GC vs CC	0.78(0.60–1.02)	0.067
		Recessive	GG vs GC-CC	1.14(0.84–1.56)	0.395
		Additive		0.94(0.79–1.12)	0.463
*CYP19A1*	rs17601876	Allele	A	1.03(0.86–1.24)	0.743
		Co-dominant (HOM)	AA vs GG	1.06(0.71–1.59)	0.771
		Co-dominant (HET)	AG vs GG	1.03(0.80–1.34)	0.816
		Dominant	AA-AG vs GG	1.04(0.81–1.33)	0.769
		Recessive	AA vs AG-GG	1.05(0.71–1.54)	0.820
		Additive		1.03(0.86–1.24)	0.744
*CYP19A1*	rs3751599	Allele	A	1.07(0.54–1.09)	0.139
		Co-dominant (HOM)	AA vs GG	0.48(0.04–5.37)	0.555
		Co-dominant (HET)	AG vs GG	0.77(0.53–1.12)	0.166
		Dominant	AA-AG vs GG	0.76(0.53–1.10)	0.146
		Recessive	AA vs AG-GG	0.50(0.05–5.55)	0.573
		Additive		0.76(0.53–1.09)	0.136
*CYP1A2*	rs762551	Allele	C	0.95(0.80–1.14)	0.595
		Co-dominant (HOM)	CC vs AA	0.82(0.56–1.20)	0.315
		Co-dominant (HET)	CA vs AA	1.14(0.87–1.50)	0.339
		Dominant	CC-CA vs AA	1.06(0.82–1.37)	0.672
		Recessive	CC vs CA-AA	0.76(0.54–1.07)	0.116
		Additive		0.95(0.79–1.14)	0.587
*CYP1A2*	rs2470890	Allele	T	1.07(0.81–1.39)	0.646
		Co-dominant (HOM)	TT vs CC	0.42(0.13–1.34)	0.140
		Co-dominant (HET)	TC vs CC	1.23(0.91–1.66)	0.182
		Dominant	TT-TC vs CC	1.15(0.86–1.55)	0.342
		Recessive	TT vs TC-CC	0.40(0.12–1.28)	0.121
		Additive		1.07(0.81–1.39)	0.646

*SNP* single nucleotide polymorphism, *OR* odds ratio, *95% CI* 95% confidence interval, *HOM* homozygous, *HET* heterozygous

*P* value was calculated by logistic regression analysis with adjustment for age and gender

*P < 0.05 indicates statistical significance

### Stratification analysis to assess the association between CYP19A1 and CYP1A2 polymorphisms and the T2DM risk

Stratification analyses on age, gender, smoking status and drinking status between T2DM patients and non-diabetic controls, as well as the disease course and the occurrence of retinopathy in T2DM patients were then carried out to further investigate the relevance between SNPs and the T2DM risk. The data were collected after adjustment of age and gender, and were summarized in Table 4. The results suggested that in the population over 59 years old, the T2DM risk was increased in AA carriers at r4646 under both co-dominant (homozygous) and recessive models (OR = 2.01, 95% CI = 1.03–3.94, *P* = 0.041 and OR = 2.10, 95% CI = 1.10–4.02, *P* = 0.026, respectively), and in GG carriers at rs6493487 under the recessive model (OR = 2.09, 95% CI = 1.07–4.08, *P* = 0.032), as well as in AA carriers at rs17601876 under the recessive model (OR = 1.86, 95% CI = 1.01–3.43, *P* = 0.048). However, rs1062033 in *CYP19A1* was found to be associated with a decreased risk of T2DM under GC genotype of co-dominant (OR = 0.50, 95% CI = 0.33–0.75, *P* = 0.001) and dominant models (OR = 0.59, 95% CI = 0.40–0.87, *P* = 0.008). Besides, CC genotype of rs762551 in gene *CYP1A2* was also found to be associated with a decreased risk of T2DM under the recessive model (OR = 0.55, 95% CI = 0.33–0.92, *P* = 0.023) but in the population less than 59 years old.

**Table 4:** Stratification analysis on *CYP19A1* and *CYP1A2* polymorphisms and T2DM risk

SNP	Subgroups	Co-dominant (HOM)		Co-dominant (HET)		Dominant		Recessive		Additive		Allele	
		OR (95% CI)	***P***	OR (95% CI)	***P***	OR (95% CI)	***P***	OR (95% CI)	***P***	OR (95% CI)	***P***	OR (95% CI)	***P***
***CYP19A1***													
rs4646	Age (≤ 59)	2.01(1.03–3.94)	0.041*	0.91(0.63–1.33)	0.633	1.05(0.73–1.50)	0.789	2.10(1.10–4.02)	0.026*	1.19(0.90–1.56)	0.226	1.20(0.92–1.58)	0.185
rs6493487	Age (≤ 59)	1.96(0.98–3.91)	0.055	0.86(0.59–1.26)	0.442	1.00(0.70–1.43)	0.997	2.09(1.07–4.08)	0.032*	1.14(0.87–1.51)	0.345	1.17(0.88–1.55)	0.280
rs1062033	Age (≤ 59)	0.93(0.55–1.57)	0.783	0.50(0.33–0.75)	0.001*	0.59(0.40–0.87)	0.008*	1.41(0.89–2.23)	0.149	0.88(0.68–1.14)	0.333	0.88(0.68–1.13)	0.319
rs17601876	Age (≤ 59)	1.79(0.94–3.40)	0.076	0.93(0.64–1.35)	0.690	1.04(0.73–1.49)	0.818	1.86(1.01–3.43)	0.048*	1.16(0.89–1.53)	0.275	1.17(0.89–1.53)	0.254
rs3751599	Diabetic retinopathy	–	–	0.53(0.27–1.04)	0.064	0.51(0.26–0.98)	0.045*	–	–	0.50(0.26–0.95)	0.034*	0.53(0.29–0.99)	0.044*
***CYP1A2***													
rs762551	Age (> 59)	0.63(0.36–1.10)	0.106	1.21(0.82–1.78)	0.344	1.06(0.73–1.54)	0.771	0.55(0.33–0.92)	0.023*	0.87(0.67–1.13)	0.299	0.88(0.69–1.12)	0.290
	BMI (≤ 24)	0.32(0.14–0.74)	0.006*	1.07(0.62–1.86)	0.799	0.81(0.49–1.36)	0.424	0.31(0.14–0.66)	0.002*	0.67(0.46–0.96)	0.031*	0.70(0.49–1.00)	0.047*
rs2470890	Disease course (> 9)	–	–	0.54(0.31–0.95)	0.033*	0.61(0.35–1.06)	0.077	–	–	0.72(0.43–1.20)	0.208	0.77(0.48–1.25)	0.290

*SNP* single nucleotide polymorphism, *OR* odds ratio, *95% CI* 95% confidence interval, *HOM* homozygous, *HET* heterozygous, *BMI* body mass index

“-” indicates no results

*P* value was calculated by logistic regression analysis with adjustment for age and gender

*P < 0.05 indicates statistical significance

Meanwhile, significant associations were found between *CYP19A1* rs3751599 and the decreased risk of retinopathy in T2DM patients under the allelic (OR = 0.53, 95% CI = 0.29–0.99, *P* = 0.044), dominant (OR = 0.51, 95% CI = 0.26–0.98, *P* = 0.045) and additive (OR = 0.50, 95% CI = 0.26–0.95, *P* = 0.034) models. The results based on individuals with body mass index (BMI) over 24 kg/m^2^ suggested that *CYP1A2* rs762551 served as a protective factor of T2DM under the allelic model (OR = 0.70, 95% CI = 0.49–1.00, *P* = 0.047), co-dominant model with CC genotype (OR = 0.32, 95% CI = 0.14–0.74, *P* = 0.006), recessive model (OR = 0.31, 95% CI = 0.14–0.66, *P* = 0.002) and additive model (OR = 0.67, 95% CI = 0.46–0.96, *P* = 0.031). The related results also suggested that carriers with heterozygous variant allele at rs2470890 of *CYP1A2* decreased 0.54-fold risk of T2DM among patients with disease course over 9 years.

### Haplotype analysis

The haplotype analysis on *CYP19A1* and *CYP1A2* polymorphisms (Table 5) results in the generation of two haplotype blocks that contains rs17601876 and rs3751599 of *CYP19A1* in block 1, and rs762551 and rs2470890 of *CYP1A2* in block 2. However, no significant association was detected between the haplotypes and T2DM risk. Furthermore, on the basis of stratification analysis results, haplotype C_rs1062033_G_rs17601876_A_rs3751599_ in *CYP19A1* was found to be associated with the decreased risk of retinopathy in T2DM patients.

**Table 5:** Association of *CYP19A1* and *CYP1A2* haplotypes with T2DM risk

Subgroup	Gene	SNP	Haplotype	Fre-case	Fre-control	OR(95% CI)	***P***
Overall	*CYP19A1*	rs17601876|rs3751599	GA	0.058	0.074	0.76(0.53–1.09)	0.136
			AG	0.341	0.334	1.03(0.86–1.24)	0.744
			GG	0.398	0.408	0.96(0.81–1.15)	0.666
	*CYP1A2*	rs762551|rs2470890	AT	0.120	0.114	1.07(0.81–1.40)	0.640
			CC	0.401	0.413	0.95(0.79–1.14)	0.586
			AC	0.478	0.473	1.02(0.86–1.21)	0.825
Diabetic retinopathy	*CYP19A1*	rs1062033|rs17601876|rs3751599	CGA	0.042	0.081	0.48(0.25–0.91)	0.026*
			CAG	0.330	0.352	0.90(0.65–1.23)	0.509
			GGG	0.429	0.409	1.05(0.79–1.41)	0.725
			CGG	0.196	0.151	1.45(0.97–2.18)	0.072

*SNP* single nucleotide polymorphism, *OR* odds ratio, *95% CI* 95% confidence interval

*P* value was calculated by logistic regression analysis with adjustment for age and gender

*P < 0.05 indicates statistical significance

## Discussion

T2DM is a complicated and multi-factorial disease, and is a serious threat to global public health. It was reported that in China there are over 100 million patients with diabetes and the prevalence rate is still on the rise [22]. Therefore, this population-based case-control study was set up, and firstly demonstrated the effects of *CYP19A1* and *CYP1A2* gene polymorphisms on the T2DM susceptibility.

The results suggested that *CYP19A1* rs1062033 was correlated with the decreased risk of T2DM, and acted as a protective factor of T2DM in patients less than 59 years old. However, by studying the the population of Chinese Han over 64 years old, Wang et al. suggested that rs1062033 might increase the risk of stroke [23]. Kopp et al. also found that rs1062033 is associated with alcohol dependence differences in estrone sulphate levels [24], but in the stratified analysis of this study, the relationship between this locus and the alcohol consumption was not observed. To the best of our knowledge, there were limited studies on *CYP19A1* rs1062033 and no related finding in diabetes has been reported to date. Therefore, based on the findings in this study, genotype GC of *CYP19A1* rs1062033 was speculated to be of protective importance in T2DM patients especially in the population less than 59 years old.

According to the database of RegulomeDB, *CYP19A1* rs3751599 and *CYP1A2* rs2470890 were assigned rank scores of 1f and 2b, respectively. These two loci were considered to locate in crucial areas of genomic function such as eQTL, TF binding sites and DNase hypersensitivity regions that may affect the protein binding and the expression of target genes. This study proved that rs3751599 was associated with a decreased risk of retinopathy in T2DM patients, and rs2470890 could also decrease the risk of T2DM in patients with disease course over 9 years. A GWAS study in Chinese Han illustrated that rs3751599 is a human height-related SNP in *CYP19A1* locus [25]. *CYP1A2* rs2470890 polymorphisms have been studied in breast cancer of northern China, and in depression of Chinese Han, as well as in pharmacogenomics analysis among Chinese [26–28]. However, neither of these two SNPs were investigated in T2DM, and this is the first study to carry out the data-based association analysis and found their protective effects on T2DM in certain populations.

In the published articles, *CYP19A1* rs4646 and *CYP1A2* rs762551 were extensively studied. Umamaheswaran et al. found that the allele and genotype frequencies of *CYP19A1* rs4646 were significantly different in South Indians and Hans Chinese in Beijing, indicating a statistically significant inter-ethnic difference of rs4646 polymorphism [29]. Therefore, it is meaningful to study the relationship between rs4646 polymorphism and the specific disease in the population of Chinese Han. *CYP19A1* rs4646 was wildly discussed in estrogen-related diseases, and the rs4646 polymorphism was believed to affect the aromatase activity and the effect of estrogen [30]. Meanwhile, De et al. highlighted the protective role of estrogen in diabetes and illustrated gender differences in the performance and outcome of diabetes based on clinical and preclinical data [31]. However, no significant association was found between gender and the rs4646 polymorphism in this study, but the relevant findings suggested that the effect of the rs4646 polymorphism on the T2DM risk was dependent on age. The rs762551 (CYP1A2*1F; −163C > A) polymorphism in intron 1 of *CYP1A2* at position 734 downstream of the first transcribed nucleotide was reported to associate with caffeine intake in different genders and ethnicities [32, 33], and to play a role in lipid metabolism thereby indicating a participation in age-related macular degeneration [34]. By studying the T2DM patients, results of this study indicated that the contribution of the rs762551 polymorphism was age-related but not gender-related. Besides, this study also found the rs762551 polymorphism was the protective factor for the population with a BMI less than 24 kg/m^2^. However, further functional study is still necessary to investigate the role of these SNPs in the risk of T2DM.

### Study strengths and limitations

This is the first study to explore the effects of *CYP19A1* and *CYP1A2* on T2DM in the population of Chinese Han, and reported that the influence of the genetic polymorphisms of *CYP19A1* and *CYP1A2* on the risk of T2DM may be related to age, BMI and disease course. However, the limitations of this study included the lack of sample size and the loss of information regarding habitual consumption of some dietary components known to affect *CYP19A1* and *CYP1A2* activities. To further explore the susceptibility loci of T2DM, larger sample collections are needed, and joint actions of environmental factors including lifestyle, dietary and climates have to be taken into consideration.

## Conclusion

To conclude, this study put forward some associations between *CYP19A1* and *CYP1A2* gene polymorphisms with the T2DM susceptibility under different genetic models, and suggested the potential role of *CYP19A1* and *CYP1A2* variations with T2DM risk among the Chinese Han population. This study provides new insights into the search for drug therapy targets, but subsequent mechanism studies are still needed to enrich the results.

## Supplementary information

**Additional file 1: Supplementary Table 1**. Sequences of oligonucleotide primers used to analysis gene polymorphisms. **Supplementary Table 2**. Frequency distributions of the allele and genotype of SNPs in *CYP19A1* and *CYP1A2.* **Supplementary Table 3**. Association analysis on clinical indexes of T2DM and *CYP19A1* rs1062033 polymorphisms

## Data Availability

All data obtained from the current study are available from the corresponding author upon reasonable request.

## Abbreviations

T2DM: Type 2 diabetes mellitus
GWAS: Genome-wide association study
SNP: Single nucleotide polymorphism
FPG: Fast plasma glucose
MAF: Minor allele frequency
LD: Linkage disequilibrium
HWE: Hardy-Weinberg equilibrium
OR: Odds ratio
CI: Confidence interval
eQTL: Expression quantitative trait loci
TF: Transcription factor
BMI: Body mass index
