# Supplementary material for: Association of CYP19A1 and CYP1A2 genetic polymorphisms with type 2 diabetes mellitus risk in the Chinese Han population
Source: Lipids Health Dis. 2020 Aug 19;19:187. doi: 10.1186/s12944-020-01366-9 (PMC7437009; doi:10.1186/s12944-020-01366-9)
Supplement: Supplementary file 1 — Additional file 1: Supplementary Table 1. Sequences of oligonucleotide primers used to analysis gene polymorphisms. Supplementary Table 2. Frequency distributions of the allele and genotype of SNPs in CYP19A1 and CYP1A2. Supplementary Table 3. Association analysis on clinical indexes of T2DM and CYP19A1 rs1062033 polymorphisms [file 12944_2020_1366_MOESM1_ESM.docx]

Supplementary Table 1: Sequences of oligonucleotide primers used to analysis gene polymorphisms

| SNP | 1st-PCRP | 2nd-PCRP | UEP_DIR | UEP |
| --- | --- | --- | --- | --- |
| rs4646 | ACGTTGGATGTCTCTTGTAGCCTGGTTCTC | ACGTTGGATGTACCTCCTATGGGTTGTCAC | R | gggtTCACCAAGCTAGGTGCTATT |
| rs6493487 | ACGTTGGATGTCCAGTGAATTCCATTCCTG | ACGTTGGATGAGTTCTTGAAAAGTTGTGCC | R | TTATTTGATAACTTTTTAACCTAATGA |
| rs1062033 | ACGTTGGATGAAAGCTGTTATCAGCGGTCC | ACGTTGGATGATCCTAGCATGTGGAAAAGC | R | TGGAAAAGCTCCCTGA |
| rs17601876 | ACGTTGGATGACTGACCACTTGACAAGTGC | ACGTTGGATGCCTCTCTGAAACTCTCCTTG | R | ACTCTCCTTGACTACCC |
| rs3751599 | ACGTTGGATGACTGGCTGAGCTTCTACTTG | ACGTTGGATGGACTAGGTAAGATTAGAGGC | F | gTAGAGGCTATACCAGATGTTT |
| rs762551 | ACGTTGGATGGAATCTTGAGGCTCCTTTCC | ACGTTGGATGCTAAGCTCCATCTACCATGC | R | CTACCATGCGTCCTG |
| rs2470890 | ACGTTGGATGGCCTCAGAATGGTGGTGTCT | ACGTTGGATGTCTACGGGCTGACCATGAAG | F | CTGCGCTTCTCCATCAA |

SNP, single nucleotide polymorphism; PCRP, polymerase chain reaction primer; UEP_DIR, unique base extension primer direction; F, forward; R, reverse; UEP, unique base extension primer.

Sequences are written in the 5'-3' (left to right) orientation.

Supplementary Table 2 Frequency distributions of the allele and genotype of SNPs in *CYP19A1* and *CYP1A2*

| Gene | SNP | Group | Allele | | Genotype | | |
| --- | --- | --- | --- | --- | --- | --- | --- |
|  |  |  | A | B | AA | AB | BB |
| *CYP19A1* | rs4646 | case | 322 (31.4%) | 702 (66.1%) | 56 (10.9%) | 210 (41.0%) | 246 (48.0%) |
|  |  | control | 299 (29.0%) | 727 (70.6%) | 41 (8.0%) | 217 (42.1%) | 255 (49.5%) |
| *CYP19A1* | rs6493487 | case | 295 (28.8%) | 729 (71.2%) | 50 (9.8%) | 195 (38.1%) | 267 (52.1%) |
|  |  | control | 271 (26.3%) | 753 (73.1%) | 34 (6.6%) | 203 (39.4%) | 275 (53.4%) |
| *CYP19A1* | rs1062033 | case | 441 (43.1%) | 581 (56.7%) | 104 (20.3%) | 233 (45.5%) | 174 (34.0%) |
|  |  | control | 461 (44.8%) | 569 (55.2%) | 94 (18.3%) | 273 (53.0%) | 148 (28.7%) |
| *CYP19A1* | rs17601876 | case | 349 (34.1%) | 675 (65.9%) | 60 (11.7%) | 229 (44.7%) | 223 (43.6%) |
|  |  | control | 344 (33.4%) | 686 (66.6%) | 58 (11.3%) | 228 (44.3%) | 229 (44.5%) |
| *CYP19A1* | rs3751599 | case | 59 (5.8%) | 965 (94.2%) | 1 (0.2%) | 57 (11.1%) | 454 (88.7%) |
|  |  | control | 76 (7.4%) | 954 (92.6%) | 2 (0.4%) | 72 (14.0%) | 441 (85.6%) |
| *CYP1A2* | rs762551 | case | 412 (40.2%) | 612 (59.8%) | 70 (13.7%) | 272 (53.1%) | 170 (33.2%) |
|  |  | control | 423 (41.1%) | 599 (58.2%) | 88 (17.1%) | 247 (48.0%) | 176 (34.2%) |
| *CYP1A2* | rs2470890 | case | 124 (12.1%) | 900 (87.9%) | 4 (0.8%) | 116 (22.7%) | 392 (76.6%) |
|  |  | control | 118 (11.5%) | 912 (88.5%) | 10 (1.9%) | 98 (19.0%) | 407 (79.0%) |

SNP, single nucleotide polymorphism.

Supplementary Table 3 Association analysis on clinical indexes of T2DM and *CYP19A1* rs1062033 polymorphisms

| SNP | | FPG (mmol/L) | GHb (%) | TCHO (mmol/L) | TG (mmol/L) | LDL (mmol/L) | HDL (mmol/L) |
| --- | --- | --- | --- | --- | --- | --- | --- |
| rs1062033 | CC | 10.18 ± 5.27 | 9.08 ± 2.10 | 4.54 ± 1.11 | 2.29 ± 2.00 | 2.71 ± 0.91 | 1.19 ± 0.60 |
|  | GC | 9.75 ± 4.01 | 9.41 ± 2.16 | 4.65 ± 1.41 | 2.61 ± 2.48 | 2.81 ± 0.99 | 1.25 ± 0.67 |
|  | GG | 9.98 ± 5.01 | 9.44 ± 3.48 | 4.68 ± 1.43 | 2.58 ± 2.15 | 2.77 ± 0.93 | 1.20 ± 0.65 |
|  | *P* | 0.769 | 0.516 | 0.735 | 0.529 | 0.722 | 0.747 |
| SNP | | BUN (mmol/L) | CRE (umol/L) | Cys-C (mg/L) | INS (μU/mL) | GFR (ml/min) | CP (ng/mL) |
| rs1062033 | CC | 6.58 ± 4.53 | 63.23 ± 20.34 | 1.19 ± 3.61 | 20.22 ± 21.88 | 120.72 ± 33.53 | 1.35 ± 0.97 |
|  | GC | 6.27 ± 2.71 | 61.80 ± 17.48 | 0.86 ± 0.57 | 18.23 ± 18.14 | 126.64 ± 38.13 | 1.39 ± 2.12 |
|  | GG | 6.28 ± 2.00 | 66.60 ± 23.62 | 0.82 ± 0.21 | 17.26 ± 11.77 | 117.54 ± 34.65 | 1.37 ± 0.75 |
|  | *P* | 0.747 | 0.282 | 0.429 | 0.547 | 0.213 | 0.981 |

SNP, single nucleotide polymorphism; FPG, fasting plasma glucose; GHb, glycated hemoglobin; TCHO, total cholesterol; TG, triglyceride; LDL, low density lipoprotein; HDL, high-density lipoprotein; BUN, blood urea nitrogen; CRE, creatinine; Cys-C, cystatin C; INS, insulin; GFR, glomerular filtration rate; CP, C-peptide.
